# Exploring the association between dietary caffeine and chronic musculoskeletal pain: a cross-sectional analysis of NHANES

**DOI:** 10.3389/fnut.2025.1570403

**Published:** 2025-04-28

**Authors:** Zhiqiang Liao, Junjian Zeng, Yixun Chen, Zhonghua Liu, Zhidong Zhou

**Affiliations:** ^1^Department of Anesthesiology, The Second Affiliated Hospital, Jiangxi Medical College, Nanchang University, Nanchang, China; ^2^Jiangxi Province Key Laboratory of Anesthesiology, Nanchang, China

**Keywords:** caffeine, chronic musculoskeletal pain, logistic regression, restricted cubic spline regression, sensitivity analysis, NHANES, cross-sectional study

## Abstract

**Background:**

The association between dietary caffeine intake and chronic musculoskeletal pain (CMP) remains unclear, with previous studies yielding conflicting results. This study aims to investigate the association between dietary caffeine intake and CMP.

**Methods:**

This cross-sectional study utilized data from the 2009–2010 National Health and Nutrition Examination Survey (NHANES) in the United States. We employed multivariable logistic regression models, restricted cubic spline regression (RCS), stratified analysis, and sensitivity analysis to evaluate the association between dietary caffeine intake and CMP.

**Results:**

The study comprised 3,797 participants, with a mean age of 50.11 ± 17.57 years and a CMP prevalence of 18.41%. After full adjustment, multivariable logistic regression and RCS regression indicated a linear positive correlation between dietary caffeine intake and CMP. For each one-unit increase in log-transformed dietary caffeine intake, the risk of CMP increased by 8.35% (OR: 1.0835, 95% CI: 1.0351, 1.1358). Compared with the Q1 (−1.00–5.44 mg/d), the ORs for individuals in the Q2 (5.45–6.83 mg/d), Q3 (6.84–7.85 mg/d), and Q4 (7.86–11.48 mg/d) were 1.1556 (95% CI: 0.8866, 1.5075, *p* = 0.2852), 1.4256 (95% CI: 1.1006, 1.8505, *p* = 0.0074), and 1.5238 (95% CI: 1.1685, 1.9920, *p* = 0.0020), respectively. Additionally, stratified and sensitivity analyses yielded similar results.

**Conclusion:**

The study revealed a positive relationship between dietary caffeine intake and CMP, suggesting that higher caffeine consumption may be linked to an increased risk of CMP. Based on these findings, CMP patients may benefit from reducing their caffeine intake.

## 1 Introduction

Chronic musculoskeletal pain (CMP) is defined as primary or secondary pain caused by bones, joints, muscles, or associated soft tissues, with a duration of 3 months or more. The most common types of CMP include chronic low back pain, neck pain, osteoarthritis of the hip and knee joints, and fibromyalgia (1). The World Health Organization (WHO) reports that around 1.75 billion people worldwide (approximately 20–33%) endure different forms of CMP. The prevalence of various types of CMP differs widely, with low back pain affecting 30–40% of adults, neck and shoulder pain affecting 15–20%, and fibromyalgia and rheumatoid arthritis representing only 2% (2). CMP not only induces pain stimuli in patients but also has the potential to cause depression, anxiety, and sleep disturbances, which in turn exacerbate the pain experience (3). Furthermore, CMP affects brain aging, leading to a decline in cognitive function and an increased risk of dementia (4). Recent research reports have highlighted that CMP is strongly linked to the onset and development of cardiovascular metabolic diseases (including diabetes, stroke, and heart disease) in middle-aged and elderly individuals, and it has been recommended for inclusion in primary and secondary prevention management of multimorbidity in this population (5).

Dietary nutrition is not only an important aspect of daily life but also a major modifiable determinant of chronic diseases (6). Nutrition, as an important component of musculoskeletal health, plays a supportive role in muscle, bone structure, and immune regulation (7). Essential fatty acids, such as arachidonic acid and tryptophan, which can only be obtained from food, serve as components of the body’s endogenous pain control system (8). Moreover, various types of CMP are related to certain dietary nutrients. Omega-3 polyunsaturated fatty acid deficiency is associated with arthritis, and supplementation with omega-3 fatty acids can help alleviate arthritis pain and reduce analgesic drug use (9). Insufficient selenium intake is associated with the severity of fibromyalgia (10). Some pro-inflammatory diets contribute to an increased risk of knee osteoarthritis and other pain-related symptoms (11). Caffeine, as one of the most popular beverages globally, is consumed daily by around 64% of American adults. The average caffeine intake for coffee drinkers is 233 mg/day, compared to 72.3 mg/day for non-coffee drinkers (12). However, the results of studies on the relationship between dietary caffeine intake and CMP are currently inconsistent (13). Therefore, we conducted a cross-sectional study based on the National Health and Nutrition Examination Survey (NHANES) to assess the association between dietary caffeine intake and CMP.

## 2 Methods

### 2.1 Study population

The NHANES project is a study conducted by the centers for disease control and prevention (CDC) involving the entire U.S. population. After obtaining a nationally representative cohort of nearly 5,000 people through stratified, multistage probability sampling, trained interviewers conducted demographic assessments, laboratory tests, and comprehensive interviews with participants, covering gender, age, race, PIR, clinical profiles, and personal behaviors (14). The NHANES survey is authorized by the national center for health statistics (NCHS) institutional review board, and as such, no further institutional review board approval is required for this secondary analysis. Additional information on NHANES can be found on its website^1^.

**Figure 1:**
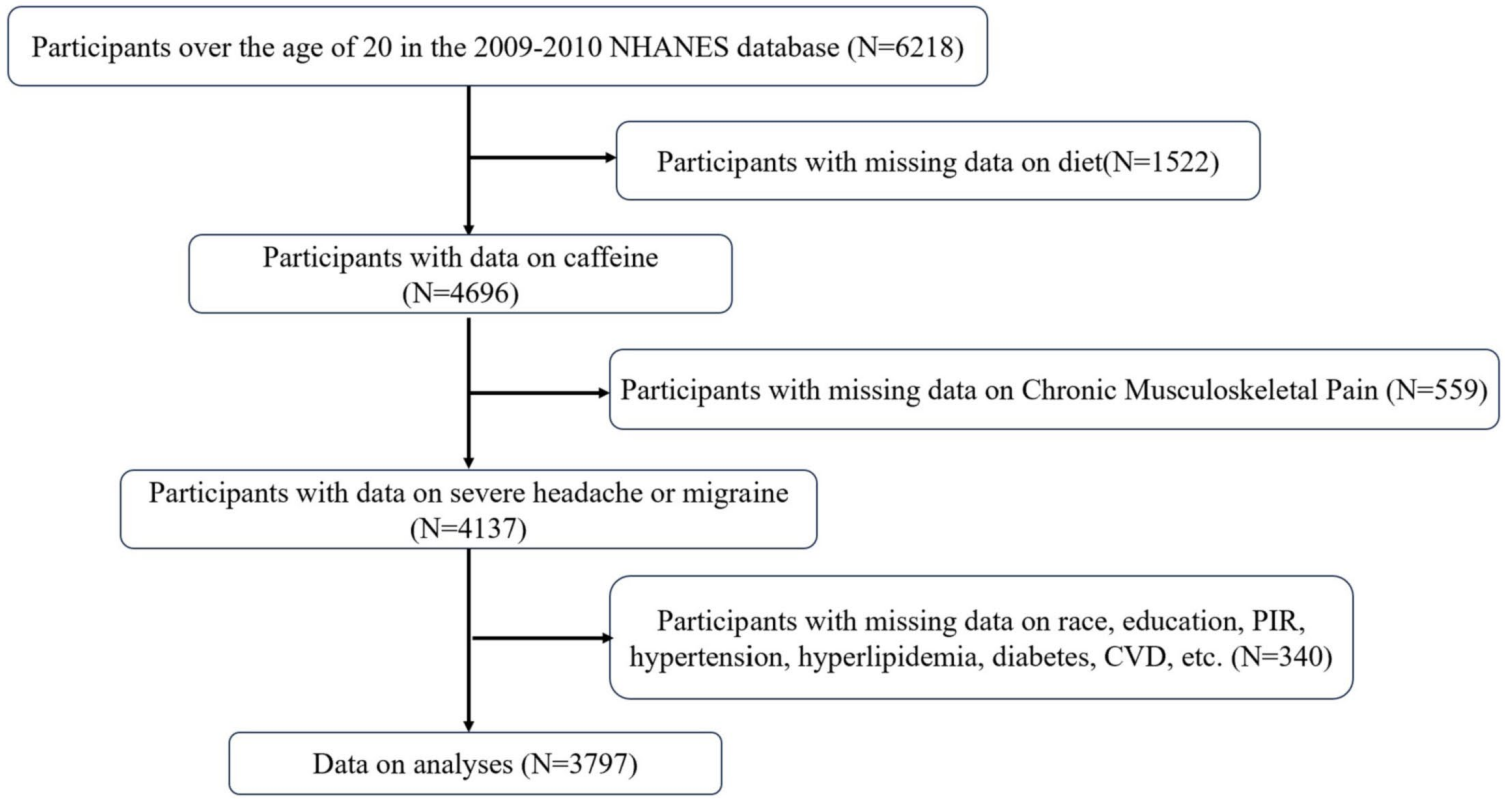
Flowchart of the study participant.

This cross-sectional study utilized NHANES data from 2009 to 2010. It included individuals with fully available CMP-related data, accurate dietary recall, and relevant confounding variables (*n* = 3,797). Of the initial 6,218 participants aged 20 years or older, 1,522 were excluded due to missing dietary recall data. Furthermore, 559 participants with missing CMP-related data and 340 participants lacking sufficient covariate information were also excluded. Figure 1 depicts the detailed process of participant exclusion and inclusion.

### 2.2 Dietary caffeine intake

Participants participated in two 24-h dietary recall interviews, during which they recalled all foods and beverages consumed from midnight to midnight of the previous day. The first interview took place at the Mobile Examination Center (MEC), while the second interview was conducted via phone 3–10 days after the first interview, covering both weekdays and weekends (15). The caffeine intake (mg/day) was estimated by trained personnel using the United States Department of Agriculture’s Food and Nutrition Database for Dietary Studies (16). Furthermore, the database contains more than 50 types of coffee/coffee beverages, 30 types of tea, and both caffeinated and non-caffeinated sodas (17). Therefore, caffeine intake was estimated based on all caffeinated dietary products. We averaged the dietary caffeine obtained from both interviews, took the logarithm, and then divided it into four groups based on quartiles (Q): Q1 (−1.00–5.44 mg/d); Q2 (5.45–6.83 mg/d); Q3 (6.84–7.85 mg/d); Q4 (7.86–11.48 mg/d). The dataset does not include dietary supplements.

### 2.3 Chronic musculoskeletal pain assessment

CMP is defined as pain that persists or recurs for more than 3 months. Pain-related questions from the arthritis survey questionnaire (ARQ) interview data were used. Participants who reported experiencing “neck pain,” “upper back pain,” “mid-back pain,” “lower back pain,” “hip pain,” or “rib pain” for 3 months or more were categorized into the CMP group.

### 2.4 Covariates

To minimize bias, based on clinical experience and existing literature, we collected relevant potential variables including gender (male or female), age (20 years and older), race (mexican american, other hispanic, non-hispanic white, non-hispanic black, or other races), education level (less than high school, high school or equivalent, and more than high school), marital status (married, unmarried, widowed, separated, divorced, or living with a partner), poverty-to-income ratio (PIR) (<1.3, 1.3 ≤ PIR < 3.5, or ≥3.5), body mass index (BMI) (<20, 20 ≤ BMI < 25, 25 ≤ BMI < 30, ≥30), smoking status (assessed by serum cotinine), drinking status (defined as drinking if consuming at least 12 alcoholic beverages per year), physical recreational activity (none, moderate, vigorous), and diagnoses of hypertension, diabetes, and cardiovascular diseases (CVD) (congestive heart failure, coronary artery disease, angina, heart attack, and stroke) based on self-report in the survey. Sleep time was derived from the participants’ usual sleep patterns during the daytime or nighttime on weekdays. In addition, the patient health questionnaire (PHQ-9) was used to assess the frequency of depressive symptoms over the past 2 weeks (18). The PHQ-9 consists of 10 questions, and a total score ranging from 0 to 27 can be calculated for individuals who fully answered 9 of the depression screening tools. A total score of ≥10 for each participant was classified as depression (19). Moreover, those managing CMP with ibuprofen, naproxen, indomethacin, aspirin, and selective COX-2 inhibitors were classified as using nonsteroidal anti-inflammatory drugs (NSAIDs).

### 2.5 Statistical analysis

In this study, categorical variables were represented as percentages, and continuous variables were expressed as mean ± standard deviation (SD). To describe the differences between groups, one-way analysis of variance was used for continuous variables, and chi-square tests were employed for categorical variables. Dietary caffeine intake was non-normally distributed, expressed in mg/day, and log-transformed to approximate a normal distribution. To evaluate the relationship between dietary caffeine intake and CMP, multiple logistic regression models were employed, constructing four models while controlling for relevant covariates: Model 1, which was unadjusted; Model 2, adjusted for sex, age, race, marital status, education level, PIR, and BMI; Model 3, further adjusted for alcohol consumption, serum cotinine, physical activity, hypertension, diabetes, CVD, sleep duration, and depression; and Model 4, fully adjusted for all covariates (Model 3 covariates plus total dietary energy, protein, fat, and carbohydrates). Dietary caffeine intake was categorized into quartiles, with the first quartile serving as the reference to examine potential non-linear relationships. Additionally, RCS were applied to explore the dose–response relationship. Subgroup analyses were performed according to sex, age (20–39, 40–59, ≥60 years), race, BMI, PIR, diabetes, hypertension, CVD, and other factors to assess heterogeneity among subgroups. Sensitivity analyses were conducted to assess the robustness of the results, including multiple imputation of missing covariate data, removing caffeine intake outliers using boxplots, and adjusting for NSAIDs to investigate their potential impact on the outcome. Statistical analysis was performed using DecisionLink 1.0, with *p* < 0.05 considered statistically significant.

## 3 Results

### 3.1 Basic characteristics of the study population

Among the 3,797 participants in the study, the average age was 50.11 ± 17.57 years, and the prevalence of CMP was 18.41%, with 51.41% of participants being female. The results indicated that older adults, males, non-Hispanic whites, married individuals, those with obesity, higher income and education levels, those consuming more than 12 alcoholic beverages annually, those with higher serum cotinine levels, and those with lower levels of physical activity had higher dietary caffeine intake. Furthermore, higher dietary caffeine intake was associated with lower incidence of hypertension, diabetes, and CVD, as well as higher intake of energy, protein, carbohydrates, and fats. The relevant baseline characteristics are presented in Table 1.

**Table 1:** Selected characteristics of NHANES 2009–2010 participants (*N* = 3,797).

Characteristic	Total	Caffeine intake (mg/d)				*p*-value
		Q1 (−1.00–5.44)	Q2 (5.45–6.83)	Q3 (6.84–7.85)	Q4 (7.86–11.48)	
No.	3,797	951	949	952	945	
Age (years, mean ± SD)	50.11 ± 17.57	49.86 ± 19	47.98 ± 18.56	50.55 ± 16.86	52.07 ± 15.39	**<0.001**
Gender, *n* (%)						**<0.001**
Female	1952 (51.41)	551 (57.94)	526 (55.43)	462 (48.53)	413 (43.70)	
Male	1845 (48.59)	400 (42.06)	423 (44.57)	490 (51.47)	532 (56.30)	
Race, *n* (%)						**<0.001**
Mexican American	642 (16.91)	209 (21.98)	198 (20.86)	151 (15.86)	84 (8.89)	
Other Hispanic	342 (9.01)	88 (9.25)	109 (11.49)	94 (9.87)	51 (5.40)	
Non-Hispanic White	2076 (54.67)	392 (41.22)	404 (42.57)	552 (57.98)	728 (77.04)	
Non-Hispanic Black	571 (15.04)	222 (23.34)	177 (18.65)	119 (12.50)	53 (5.61)	
Other race	166 (4.37)	40 (4.21)	61 (6.43)	36 (3.78)	29 (3.07)	
Educational level, *n* (%)						**<0.001**
Less than high school	934 (24.60)	265 (27.87)	267 (28.13)	200 (21.01)	202 (21.38)	
High school	873 (22.99)	216 (22.71)	210 (22.13)	217 (22.79)	230 (24.34)	
More than high school	1990 (52.41)	470 (49.42)	472 (49.74)	535 (56.20)	513 (54.29)	
Marital status, *n* (%)						**<0.001**
Married	2074 (54.62)	507 (53.31)	461 (48.58)	533 (55.99)	573 (60.63)	
Widowed	313 (8.24)	95 (9.99)	84 (8.85)	80 (8.40)	54 (5.71)	
Divorced	417 (10.98)	88 (9.25)	97 (10.22)	102 (10.71)	130 (13.76)	
Separated	112 (2.95)	26 (2.73)	29 (3.06)	30 (3.15)	27 (2.86)	
Never married	591 (15.56)	171 (17.98)	188 (19.81)	134 (14.08)	98 (10.37)	
Living with partner	290 (7.64)	64 (6.73)	90 (9.48)	73 (7.67)	63 (6.67)	
PIR, *n* (%)						**<0.001**
< 1.3	1,161 (30.58)	301 (31.65)	329 (34.67)	264 (27.73)	267 (28.25)	
1.3 ≤ PIR < 3.5	1,429 (37.63)	383 (40.27)	378 (39.83)	356 (37.39)	312 (33.02)	
≥3.5	1,207 (31.79)	267 (28.08)	242 (25.50)	332 (34.87)	366 (38.73)	
BMI, *n* (%)						0.068
< 20	150 (3.95)	36 (3.79)	49 (5.16)	33 (3.47)	32 (3.39)	
20 ≤ BMI < 25	869 (22.89)	235 (24.71)	206 (21.71)	236 (24.79)	192 (20.32)	
25 ≤ BMI < 30	1,287 (33.90)	313 (32.91)	322 (33.93)	333 (34.98)	319 (33.76)	
≥30	1,491 (39.27)	367 (38.59)	372 (39.20)	350 (36.76)	402 (42.54)	
Cotinine (ng/mL), mean ± SD	54.19 ± 122.31	28.89 ± 89	41.51 ± 108.65	56.64 ± 124.93	89.89 ± 150.07	**<0.001**
Alcohol, *n* (%)						**<0.001**
No	980 (25.81)	327 (34.38)	290 (30.56)	194 (20.38)	169 (17.88)	
Yes	2,817 (74.19)	624 (65.62)	659 (69.44)	758 (79.62)	776 (82.12)	
Hypertension, *n* (%)						**0.007**
No	2,428 (63.95)	574 (60.36)	641 (67.54)	621 (65.23)	592 (62.65)	
Yes	1,369 (36.05)	377 (39.64)	308 (32.46)	331 (34.77)	353 (37.35)	
Diabetes, *n* (%)						0.602
No	3,357 (88.41)	832 (87.49)	848 (89.36)	845 (88.76)	832 (88.04)	
Yes	440 (11.59)	119 (12.51)	101 (10.64)	107 (11.24)	113 (11.96)	
CVD, *n* (%)						0.623
No	3,377 (88.94)	842 (88.54)	849 (89.46)	854 (89.71)	832 (88.04)	
Yes	420 (11.06)	109 (11.46)	100 (10.54)	98 (10.29)	113 (11.96)	
Physical activity, *n* (%)						0.148
None	2009 (52.91)	501 (52.68)	530 (55.85)	482 (50.63)	496 (52.49)	
Moderate	1,543 (40.64)	394 (41.43)	352 (37.09)	401 (42.12)	396 (41.90)	
Vigorous	245 (6.45)	56 (5.89)	67 (7.06)	69 (7.25)	53 (5.61)	
Total caloric intake (Kcal, mean ± SD)	2058.54 ± 818.83	1918.86 ± 793.14	1963.11 ± 761.93	2101.16 ± 838.68	2252.01 ± 838.64	**<0.001**
Total protein intakes (GM, mean ± SD)	80.82 ± 34.22	77.41 ± 33.93	77.09 ± 32.95	81.93 ± 33.34	86.85 ± 35.73	**<0.001**
Total fatty acid intake (GM, mean ± SD)	76.34 ± 37.54	68.62 ± 34.36	71.25 ± 34.57	78.62 ± 37.99	86.94 ± 40.25	**<0.001**
Total carbohydrate intake (GM, mean ± SD)	253.79 ± 105.55	242.12 ± 104.10	247.45 ± 97.18	256.12 ± 107.83	269.56 ± 110.74	**<0.001**
Sleep time (hours, mean ± SD)	6.85 ± 1.42	6.83 ± 1.44	6.96 ± 1.44	6.87 ± 1.39	6.75 ± 1.42	**0.029**
Depression, *n* (%)						**0.038**
No	3,443 (90.68)	865 (90.96)	871 (91.78)	872 (91.60)	835 (88.36)	
Yes	354 (9.32)	86 (9.04)	78 (8.22)	80 (8.40)	110 (11.64)	
NSAIDs						**<0.001**
No	2,990 (78.75)	772 (81.18)	774 (81.56)	750 (78.78)	694 (73.44)	
Yes	807 (21.25)	179 (18.82)	175 (18.44)	202 (21.22)	251 (26.56)	
Chronic pain, *n* (%)						**<0.001**
No	3,098 (81.59)	817 (85.91)	800 (84.30)	764 (80.25)	717 (75.87)	
Yes	699 (18.41)	134 (14.09)	149 (15.70)	188 (19.75)	228 (24.13)	

PIR, poverty-to-income ratio; BMI, body mass index; CVD, cardiovascular disease; GM, milligram; Kcal, kilocalorie; NSAIDs, nonsteroidal anti-inflammatory drugs; Statistically significant results (*p* < 0.05) are highlighted in bold.

### 3.2 Association between dietary caffeine intake and chronic musculoskeletal pain

In the multivariate logistic regression (Table 2), dietary caffeine intake was studied as both a continuous and categorical variable. After adjusting for all covariates, a positive correlation was observed between dietary caffeine intake and CMP when caffeine intake was considered as a continuous variable (OR: 1.0835, 95% CI: 1.0351, 1.1358, *p* = 0.0007). When dietary caffeine intake was divided into quartiles for analysis, this association remained consistent. Compared to individuals in the Q1 of caffeine intake, the ORs for individuals in Q2, Q3, and Q4 were 1.1556 (95% CI: 0.8866, 1.5075, *p* = 0.2852), 1.4256 (95% CI: 1.1006, 1.8505, *p* = 0.0074), and 1.5238 (95% CI: 1.1685, 1.9920, *p* = 0.0020), respectively.

**Table 2:** Association of caffeine with chronic musculoskeletal pain among NHANES survey participants 2009–2010.

	Model 1		Model 2		Model 3		Model 4	
Variable (mg/d)	OR (95%) CI	*p*-value	OR (95%) CI	*p*-value	OR (95%) CI	*p*-value	OR (95%) CI	*p*-value
Caffeine*	1.1377 (1.0907, 1.1882)	**<0.0001**	1.1292 (1.0801, 1.1822)	**<0.0001**	1.0879 (1.0395, 1.1401)	**0.0003**	1.0835 (1.0351, 1.1358)	**0.0007**
Caffeine* (quartile)								
Q1 (−1.00–5.44)	Reference		Reference		Reference		Reference	
Q2 (5.45–6.83)	1.1356 (0.8820, 1.4632)	0.3245	1.1050 (0.8546, 1.4297)	0.4466	1.1616 (0.8916, 1.5147)	0.2676	1.1556 (0.8866, 1.5075)	0.2852
Q3 (6.84–7.85)	1.5003 (1.1783, 1.9147)	**0.0010**	1.5279 (1.1916, 1.9636)	**0.0009**	1.4389 (1.1119, 1.8660)	**0.0058**	1.4256 (1.1006, 1.8505)	**0.0074**
Q4 (7.86–11.48)	1.9388 (1.5339, 2.4587)	**<0.0001**	1.8633 (1.4506, 2.4010)	**<0.0001**	1.5582 (1.1978, 2.0321)	**0.0010**	1.5238 (1.1685, 1.9920)	**0.0020**
*p* for trend	**<0.0001**		**<0.0001**		**0.0004**		**0.0007**	

CI: confidence interval; OR, odds ratio.

Model 1: Unadjusted.

Model 2: Adjust for gender, age, race, educational level, marital status, PIR, BMI.

Model 3: Adjust for the variables in model 2 plus cotinine, alcohol consumption, physical activity, hypertension, diabetes, CVD, sleep time, depression.

Model 4: Adjust for the variables in model 3 plus total dietary energy, total protein, total fat, total carbohydrate.

PIR, poverty-to-income ratio; BMI, body mass index; CVD, cardiovascular disease.

Statistically significant results (*p* < 0.05) are highlighted in bold. *indicates caffeine values after logarithmic transformation.

### 3.3 Restricted cubic spline plot

**Figure 2:**
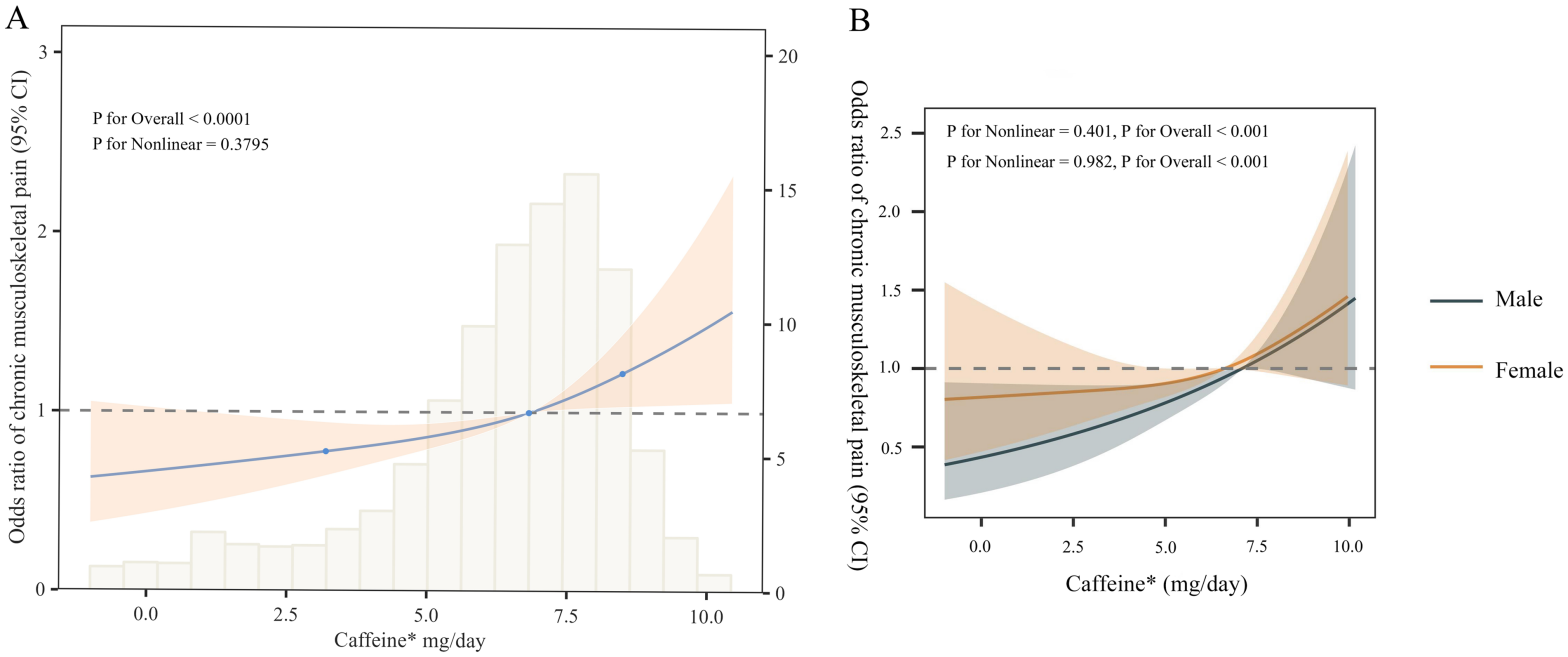
Dose–response relationship between dietary caffeine intake and chronic musculoskeletal pain in the general population **(A)** and by sex **(B)**. Adjust according to covariates of model 4.

According to the RCS analysis (Figure 2A), dietary caffeine intake is linearly positively correlated with CMP (P for non-linearity = 0.3795). Moreover, this association remains consistent across different genders (Figure 2B).

### 3.4 Interaction test and subgroup analysis

**Figure 3:**
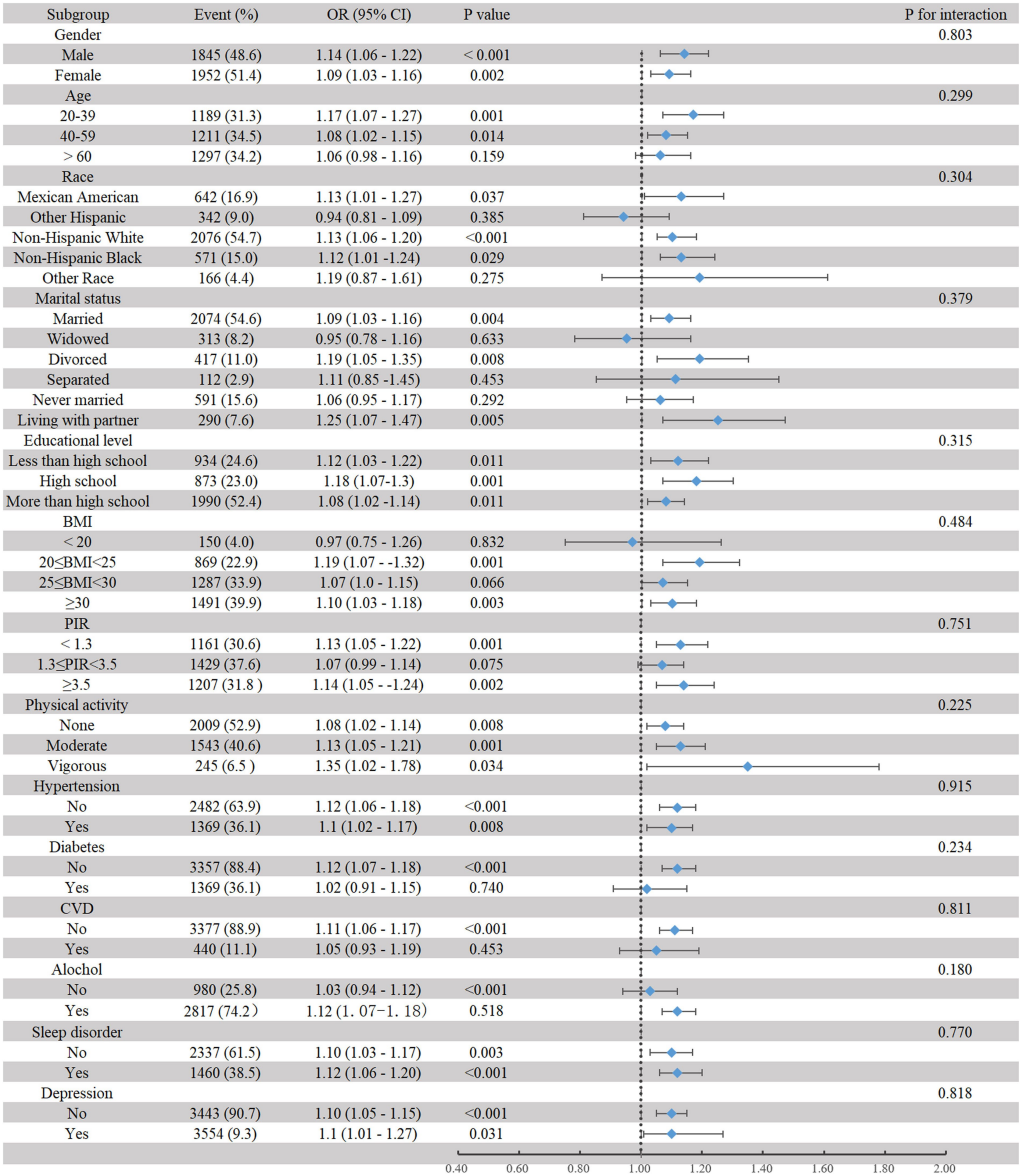
Subgroup analysis of the association between dietary caffeine intake and CMP.

No significant differences between subgroups were found in terms of gender, age, race, marital status, BMI, PIR, physical activity, hypertension, diabetes, CVD, alcohol consumption, sleep disorders, and depression (Figure 3). This indicates a positive linear relationship between dietary caffeine intake and CMP, which remains consistent across subgroups.

### 3.5 Sensitivity analysis

In Figure 1, we removed missing data. To prevent bias caused by the deletion of missing data, we retained the missing data in the sensitivity analysis and performed multiple imputations. In Supplementary Table S1, 4,137 participants were included in the study. With each unit change in log-transformed dietary caffeine intake, the risk of CMP increased by 7.33% (OR: 1.0733, 95% CI: 1.0267, 1.1233). Participants in the highest quartile Q4 of log-transformed dietary caffeine intake had a higher risk of CMP than those in the lowest quartile Q1 (OR: 1.5273, 95% CI: 1.1822, 1.9775). Furthermore, to prevent outliers in dietary caffeine from biasing the results, we removed extreme values using the Boxplot method and found that the relationship remained stable (Supplementary Table S2). The results showed that with each unit change in log-transformed dietary caffeine intake, the risk of CMP increased by 10.10% (OR: 1.1010, 95% CI: 1.0366, 1.1708). To adjust for the confounding effect of NSAID use on the outcome, we included NSAID use as a covariate in the study (Supplementary Table S3). The results showed that participants in the highest quartile of dietary caffeine intake had a 54.82% higher risk of CMP compared to those in the lowest quartile (OR: 1.5482, 95% CI: 1.0736, 2.2382).

## 4 Discussion

In this cross-sectional study using data from a nationally representative sample of the United States, we found a linear positive association between caffeine intake (whether analyzed continuously or by quartiles) and CMP, which remained significant after controlling for various influencing factors. Based on these findings, individuals with CMP may benefit from moderating their caffeine intake.

Caffeine, as a natural methylxanthine, is primarily found in coffee, tea, chocolate, and energy drinks. After oral administration, it is completely and rapidly absorbed by the human body, with a bioavailability of 100%, and can freely pass through the blood–brain barrier (20). Caffeine’s structure is similar to that of adenosine, enabling it to competitively antagonize adenosine receptors: A1, A2 (A2A and A2B), and A3 receptors (21). Previous research has concentrated on investigating the relationship between caffeine and acute pain. Caffeine reduces antinociceptive activity by competitively inhibiting adenosine A2A receptors. Moreover, caffeine can also suppress the synthesis of leukotrienes and prostaglandins. Based on these mechanisms, caffeine could be used as an adjunct to enhance the effects of analgesics (22, 23). Compared to the use of common analgesics (acetaminophen or ibuprofen) alone, adding more than 100 mg of caffeine to the standard dose of these analgesics results in a small but significant increase in the proportion of participants who experience good relief from acute pain (approximately 5 to 10% of participants) (24). However, the results of studies on the relationship between caffeine intake and chronic pain remain inconsistent. For example, Kuzu et al. used a micro-longitudinal design to show that caffeine intake is not associated with pain intensity in patients with fibromyalgia (25). Al-Khudhairy et al. pointed out that caffeine influences sleep quality and is related to CMP (26). Specifically, clinical assessments and surveys of 139 participants found that increased caffeine intake is linked to the development of CMP-related symptoms by affecting sleep quality and lowering the pain pressure threshold (26). Other research indicates that, compared to individuals without CMP, patients with CMP have significantly higher caffeine intake. To be more specific, long-term daily consumption of more than two cups of coffee is associated with frequent neck and shoulder pain (*p* = 0.016). The mediation models revealed a bidirectional relationship between shorter sleep duration and long-term caffeine intake, which may exacerbate CMP over time (Z for mediation effect = 2.95, *p* < 0.01) (27). Furthermore, several studies have indicated that caffeine suppresses the antihyperalgesic effects produced after pain treatment by engaging adenosine receptors (A1 and A2 receptors) (28–30). This may be due to caffeine intake inducing compensatory upregulation of A2 receptors, leading to neuroinflammation or abnormal neural circuitry remodeling (31). Additionally, a recent study using resting-state functional magnetic resonance imaging (fMRI) to assess neural activity in mice with long-term caffeine intake (0.3 mg/mL caffeine for 4 weeks) found that mice in the chronic caffeine intake group exhibited enhanced neural activity in certain hippocampal regions (such as the dentate gyrus) (32), which has been shown to be associated with heightened pain sensitivity (33). In our research, multivariate logistic regression showed that the OR for CMP increased progressively from Q2 to Q4 compared to individuals with the lowest caffeine intake in Q1, and this trend was statistically significant (*p* for trend = 0.007). RCS regression further demonstrated a linear positive correlation between dietary caffeine intake and CMP (*p* for non-linearity = 0.3795), which aligns with the results from the multivariate logistic regression. Additionally, several sensitivity analyses confirmed that the linear positive correlation between the two remained consistent, and subgroup analysis did not identify any particular group that influenced this relationship.

The specific mechanism underlying the positive correlation between dietary caffeine intake and CMP needs further investigation. However, our findings are consistent with existing biological evidence: firstly, caffeine disrupts sleep structure (e.g., prolonging sleep latency, reducing slow-wave sleep, decreasing total sleep time) by increasing cortisol and activating the stress system, leading to decreased sleep quality (34). Reduced sleep quality leads to adenosine accumulation, which lowers pain threshold, while also increasing levels of pain-related molecules (such as prostaglandins and nitric oxide) and decreasing levels of pain-relieving substances (such as serotonin) (35, 36). Furthermore, caffeine inhibits the reuptake of glutamate by the EAAT3 transporter, increasing the levels of glutamate around neurons, which enhances the activity of nociceptors and exacerbates mechanical sensitization, ultimately causing peripheral pain sensitization (37). Additionally, chronic caffeine consumption alters the sensitivity of adenosine receptors (A1 and A2A receptors), weakening the inhibition of nociceptive afferent fibers (C fibers) and promoting enhanced pain signal transmission, which ultimately leads to an increase in pain perception (21). Caffeine not only influences peripheral pain sensitization and pain signal conduction, but also affects central pain sensitization. Research has indicated that chronic caffeine consumption may trigger central pain sensitization by activating microglial cells and increasing pro-inflammatory cytokines like IL-1β (31). Moreover, caffeine induces diuresis by antagonizing A1 or A2A receptors in proximal renal tubular cells and interfering with antidiuretic hormone (ADH) secretion, which in turn affects magnesium levels in the body (38). Magnesium has been demonstrated to block N-methyl-D-aspartate (NMDA) receptors in a voltage-dependent manner, preventing central pain sensitization and alleviating muscle pain (39). It is noteworthy that caffeine metabolism is significantly influenced by genetic factors, particularly related to CYP1A2 gene polymorphisms (40). Genetic differences affect the rate of caffeine metabolism in individuals (41). Specifically, slow caffeine metabolizers (with the CYP1A2 slow metabolizer genotype) are more likely to accumulate caffeine, which may amplify its negative effects on pain (42). Thus, as previously mentioned, various biological evidences indicate that dietary caffeine plays a critical role in the development and progression of CMP.

Our study has several strengths. First, the study population is derived from a large, nationally representative sample of American adults. Second, we adjusted for various potential confounding factors to reduce their impact. Third, we performed RCS regression to analyze the dose–response relationship between dietary caffeine intake and CMP. Fourth, we utilized multiple multivariate logistic regression models (including sensitivity analysis) to evaluate the relationship between dietary caffeine intake and CMP. The results across all models were consistent, demonstrating the robustness of our findings. However, certain limitations must also be acknowledged. First, this is a cross-sectional study, and we cannot make causal inferences between dietary caffeine intake and CMP. Nevertheless, we adjusted for potential confounders to ensure the accuracy of the results. Second, dietary caffeine intake was collected through a 24-h recall, which may introduce recall bias. Additionally, this study did not investigate the effects of specific caffeine-containing products (e.g., coffee, tea, chocolate) on CMP. Future studies may refine the results by accurately quantifying caffeine intake from various sources and exploring the differential impact of these products on CMP. This study observed that for each unit increase in log-transformed caffeine intake, the risk of CMP increased by 8.35%. Although this risk may be moderate at the individual level, considering the widespread caffeine consumption and the public health implications of CMP, even a small relative risk change may translate into a large number of absolute cases. Therefore, further cost-effectiveness analysis is needed to accurately guide clinical recommendations.

## 5 Conclusion

In summary, our research presents epidemiological evidence indicating a certain positive correlation between dietary caffeine consumption and CMP. Future prospective studies should further confirm these associations and investigate the potential mechanisms between caffeine intake and CMP.

## Data Availability

The original contributions presented in the study are included in the article/Supplementary material, further inquiries can be directed to the corresponding author.

## Footnotes

^1^ https://www.cdc.gov/nchs/nhanes
