# Supplementary material for: Exploring the association between dietary caffeine and chronic musculoskeletal pain: a cross-sectional analysis of NHANES
Source: Front Nutr. 2025 Apr 28;12:1570403. doi: 10.3389/fnut.2025.1570403 (PMC12066468; doi:10.3389/fnut.2025.1570403)
Supplement: Supplementary file 1 [file Table_1.docx]

**Supplementary Table S1** Sensitivity analyses for the effect of caffeine on chronic musculoskeletal pain after multiple imputations of missing values (N=4137).

| **Variable (mg/d)** | **Model 1** | | **Model 2** | | **Model 3** | | **Model 4** | | |  |  |
| --- | --- | --- | --- | --- | --- | --- | --- | --- | --- | --- | --- |
|  | OR (95%) CI | P-value | OR (95%) CI | P-value | OR (95%) CI | P-value |  | OR (95%) CI | P-value |  |  |
| Caffeine* | 1.1287  (1.0837, 1.1769) | **<0.0001** | 1.1167  (1.0699, 1.1670) | **<0.0001** | 1.0765  (1.0301, 1.1264) | **0.0012** |  | 1.0733  (1.0267, 1.1233) | **0.0020** |  |  |
| Caffeine* (quartile) | |  | | | | |  |  |  |  |  |
| Q1 (-1.00 - 5.45) | Reference | | Reference | | Reference |  |  | Reference |  |  |  |
| Q2 (5.46 - 6.77) | 1.1197  (0.8789, 1.4273) | 0.3601 | 1.1000  (0.8602, 1.4075) | 0.4475 | 1.1626  (0.9004, 1.5021) | 0.2482 |  | 1.1628  (0.9003, 1.5028) | 0.2481 |  |  |
| Q3 (6.78 - 7.81) | 1.3885  (1.0994, 1.7566) | **0.0060** | 1.3873  (1.0919, 1.7655) | **0.0075** | 1.2932  (1.0067, 1.6636) | **0.0447** |  | 1.2829  (0.9978, 1.6520) | 0.0526 |  |  |
| Q4 (7.82 - 11.48) | 1.9067  (1.5244, 2.3919) | **<0.0001** | 1.8226  (1.4343, 2.3223) | **<0.0001** | 1.5509  (1.2036, 2.0030) | **0.0007** |  | 1.5273  (1.1822, 1.9775) | **0.0012** |  |  |
| P for trend | **<0.0001** |  | **<0.0001** |  | **0.0012** |  |  | **0.0011** |  |  |  |

Abbreviations: CI: confidence interval; OR, odds ratio.

Model 1: Unadjusted;

Model 2: Adjust for gender, age, race, educational level, marital status, PIR, BMI;

Model 3: Adjust for the variables in model 2 plus cotinine, alcohol consumption, physical activity, hypertension, diabetes, CVD, sleep time, depression.

Model 4: Adjust for the variables in model 3 plus total dietary energy, total protein, total fat, total carbohydrate.

Abbreviation: PIR, poverty-to-income ratio; BMI, body mass index; CVD, cardiovascular disease.

**Supplementary Table S2** Sensitivity analyses for the effect of caffeine on chronic musculoskeletal pain after the outliers are removed using the boxplot method (N=3554).

| **Variable (mg/d)** | **Model 1** | | **Model 2** | | **Model 3** | | **Model 4** | | |  |  |
| --- | --- | --- | --- | --- | --- | --- | --- | --- | --- | --- | --- |
|  | OR (95%) CI | P-value | OR (95%) CI | P-value | OR (95%) CI | P-value |  | OR (95%) CI | P-value |  |  |
| Caffeine* | 1.1663  (1.1044, 1.2329) | **<0.0001** | 1.1547  (1.0891, 1.2256) | **<0.0001** | 1.1054  (1.0410, 1.1751) | **0.0012** |  | 1.1010  (1.0366, 1.1708) | **0.0019** |  |  |
| Caffeine* (quartile) | |  | | | | |  |  |  |  |  |
| Q1 (2.00 - 5.84) | Reference | | Reference | | Reference |  |  | Reference |  |  |  |
| Q2 (5.85 - 6.97) | 1.0368  (0.8029, 1.3392) | 0.7815 | 1.0102  (0.7787, 1.3107) | 0.9389 | 1.0733  (0.8206, 1.4045) | 0.6054 |  | 1.0677  (0.8159, 1.3978) | 0.6330 |  |  |
| Q3 (6.98 - 7.90) | 1.3292  (1.0400, 1.7016) | **0.0234** | 1.3821  (1.0724, 1.7842) | **0.0127** | 1.2965  (0.9955, 1.6910) | 0.0545 |  | 1.2906  (0.9901, 1.6849) | 0.0598 |  |  |
| Q4 (7.91 - 11.07) | 1.7933  (1.4166, 2.2767) | **<0.0001** | 1.7390  (1.3500, 2.2462) | **<0.0001** | 1.4763  (1.1313, 1.9308) | **0.0043** |  | 1.4499  (1.1085, 1.9005) | **0.0069** |  |  |
| P for trend | **<0.0001** |  | **<0.0001** |  | **0.0019** |  |  | **0.0031** |  |  |  |

Abbreviations: CI: confidence interval; OR, odds ratio.

Model 1: Unadjusted;

Model 2: Adjust for gender, age, race, educational level, marital status, PIR, BMI;

Model 3: Adjust for the variables in model 2 plus cotinine, alcohol consumption, physical activity, hypertension, diabetes, CVD, sleep time, depression.

Model 4: Adjust for the variables in model 3 plus total dietary energy, total protein, total fat, total carbohydrate.

Abbreviation: PIR, poverty-to-income ratio; BMI, body mass index; CVD, cardiovascular disease.

**Supplementary Table S3** Sensitivity analyses for the effect of caffeine on chronic musculoskeletal pain after adjusting for NSAIDs

| **Variable** | **OR (95% CI)** | ***P* value** |
| --- | --- | --- |
| Caffeine*, mg/day | 1.0820 (1.0165, 1.1528) | **0.0141** |
| **Caffeine* quartiles, mg/day** |  |  |
| Q1 (-1.00 - 5.45) | Reference |  |
| Q2 (5.46 - 6.79) | 1.3268 (0.9283, 1.8988) | 0.1212 |
| Q3 (6.80 - 7.82) | 1.6624 (1.1654, 2.3776) | **0.0052** |
| Q4 (7.83 - 11.48) | 1.5482 (1.0736, 2.2382) | **0.0196** |
| P for trend |  | **0.0014** |

**Notes**: All results adjusted for the variates in Model 4 plus NSAIDs.
